# Reduced Surgical Site Infection Risk With Overlapping Knotless Tailless Versus Buried Knot Techniques in Running Subcuticular Closure for Posterior Thoracic and Lumbar Spine Surgery

**DOI:** 10.7759/cureus.94855

**Published:** 2025-10-18

**Authors:** Saechin Kim, Easton Ryan, Philip Hanna, Valerie Kiers, John G Massoud, Serafina F Zotter, Amanda Schillinger, Mark P Cote

**Affiliations:** 1 Department of Orthopedic Surgery, Massachusetts General Hospital, Harvard Medical School, Boston, USA

**Keywords:** absorbable sutures, knotless, methods of skin closure, posterior spine surgeries, surgical site infection (ssi)

## Abstract

Background: Are small abscesses and suture reactions seen with buried knots (BK) or tails at the ends of a running subcuticular skin closure using non-barbed absorbable suture (RSAS) associated with surgical site infections (SSIs)? Would the elimination of BK and tails reduce the rate of SSI? The senior author (SK) has been using an overlapping knotless tailless (KT) RSAS method since 2013. As three spine surgeons in the same division at a tertiary referral center primarily used the BK method, we decided to compare deep SSI rates between the BK and KT methods in posterior thoracic and lumbar spine surgeries.

Methods: After IRB approval, hospital records and the research patient data registry were used to retrospectively identify consecutive posterior thoracic and lumbar surgical cases at a tertiary referral academic medical center. Inclusion criteria were open posterior thoracic and lumbar spine surgery in adult patients (age >21) and skin closure using RSAS. Exclusion criteria included previous infection in the same site, oncologic surgery, multiple procedures under the same anesthesia, and less than three months of follow-up (f/u). The KT group comprised cases closed using the overlapping KT method by the senior author (SK) from 2013 to 2023 (n=266). Power analysis suggested that more than 600 cases were needed in the BK group, comprised of cases closed using the BK method by three surgeons between 2018 and 2020 (n=613). Demographics, comorbidities, and surgical characteristics were collected. The diagnosis of SSI was made by the cultures taken and/or by the primary surgeon at the return to the operating room (ROR). We identified all patients who had ROR for deep SSI within 12 months after the index surgery. All had ROR within three months of the index surgery, had a minimum of 12 months of f/u, and were included in the study.

Results: The KT group had increased risk factors for SSI compared to the BK group, such as a higher percentage of smokers, previous surgery in the same site and instrumented fusion cases, and a greater average number of levels fused per case and case length. No differences between the groups were found for other risk factors such as age, obesity, or history of diabetes. However, the rate of deep SSI in the KT group (0.4%=1/266) was less than that in BK (2.6%=16/613); the difference was statistically significant (p=0.030). Multivariate analysis of infection risk showed that the odds ratio for BK (OR 6.44, 95% CI, 1.53 - 59.77, p=0.008) and for diabetes (OR 7.27, 95% CI, 2.79 - 20.62, p<0.001) reached statistical significance.

Conclusions: This study showed that an overlapping KT method may result in a lower rate of ROR for deep SSI in posterior thoracic and lumbar spine surgery compared to the BK method. We would recommend further studies to confirm this initial finding and to determine the optimal skin closure method in spinal surgery.

## Introduction

The surgical site infection (SSI) and/or the concern for it are the most common causes for readmission following spine surgery [1] and can result in increased costs and morbidity. Identified risk factors for SSI include older age, obesity, diabetes or smoking history, previous surgery at the same site, higher number of levels fused, instrumentation, longer operating time, and longer hospital stay [2]. The bacterial seeding that causes SSI can occur prior to (i.e., contamination during surgery) or secondarily following the skin closure. Since the latter would occur via the incision, one could surmise that the skin closure method may play an important role in determining SSI rates. However, the literature is inconclusive, i.e., a systematic review in 2018 concluded that the evidence for optimal wound closure in spine surgery is lacking because there were few studies and no significant differences were found between different skin closure methods [3].

Running subcuticular skin closure using non-barbed absorbable suture (RSAS) is often used in posterior spine surgery, and the ends of the suture can be managed by buried knots (BK) or tails/knots left outside the skin. With BK, a reaction to the BK can occasionally be seen as a small abscess at the pole of the incision. For tails/knots left outside the skin, loss of skin closure tension can be seen at the pole of the incision, as well as suture reaction at the suture exit sites. The question was whether an RSAS method with neither knots nor tails that maintains skin closure tension at the pole of the incision could help to decrease the risk for SSI.

A search of the literature for knot-free or knotless subcuticular suture found four reports using RSAS, all with the expressed goal of avoiding knots and tails without losing skin approximation [4-7]. In three reports, the methods involve the suture exiting the skin and then re-entering the skin through the same exit point to anchor the suture on each side of the incision [4-6]. In the fourth, the suture starts within the wound with a series of anchoring sutures placed within the wound in the deep dermal layer on each side of the incision [7]. Two of the studies reported good outcomes, but without comparison groups [4,7].

The senior author (SK) has been using an overlapping RSAS with neither knots nor tails method since 2013. This knotless tailless (KT) method differs from the previously reported four methods because it starts outside the wound but does not involve the suture exiting skin and re-entering through the same exit point. We believe that the overlapping portion of the method helps maintain the skin closure tension at the pole of the incision.

In the same spine division, three surgeons primarily used RSAS with BK in open posterior thoracic and lumbar spine surgeries. Our goal was to compare the rates of return to the operating room (ROR) for deep SSI between the KT and BK methods. Our null hypothesis is that the rates are similar between the two methods.

## Materials and methods

Patient population

The study was conducted at the Department of Orthopedic Surgery, Massachusetts General Hospital, Harvard Medical School, Boston, MA. After receiving IRB approval, billing and hospital records, including the research patient data registry, were used to retrospectively obtain consecutive posterior thoracic and lumbar surgeries performed by four spine surgeons in a spine division of a tertiary referral center. Mass General Brigham IRB issued the approval (2021P003069).

**Figure 1:**
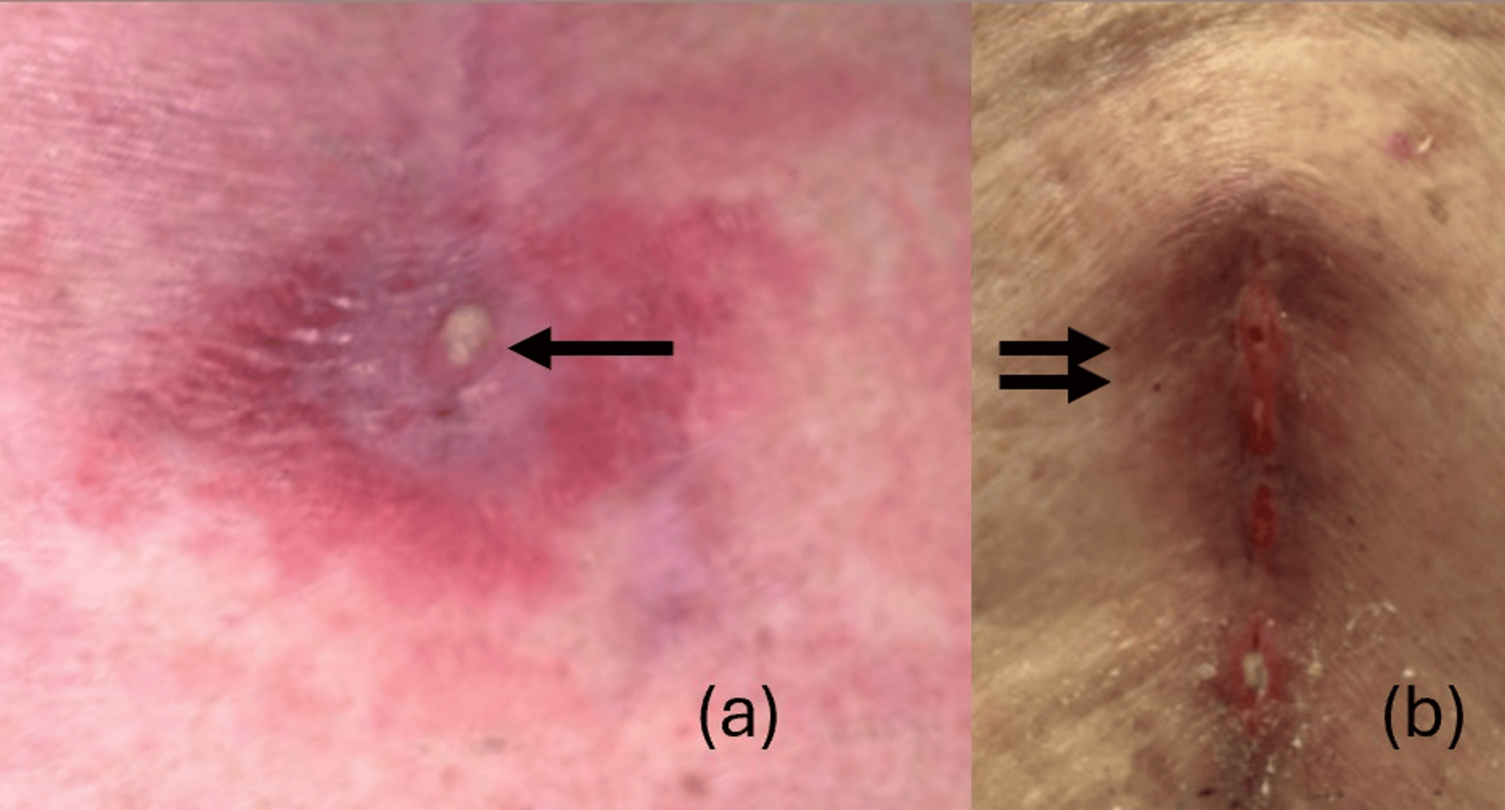
Small abscess at the pole of the incision (a) can progress to involve the whole incision (b) (marked by arrows)

**Figure 2:**
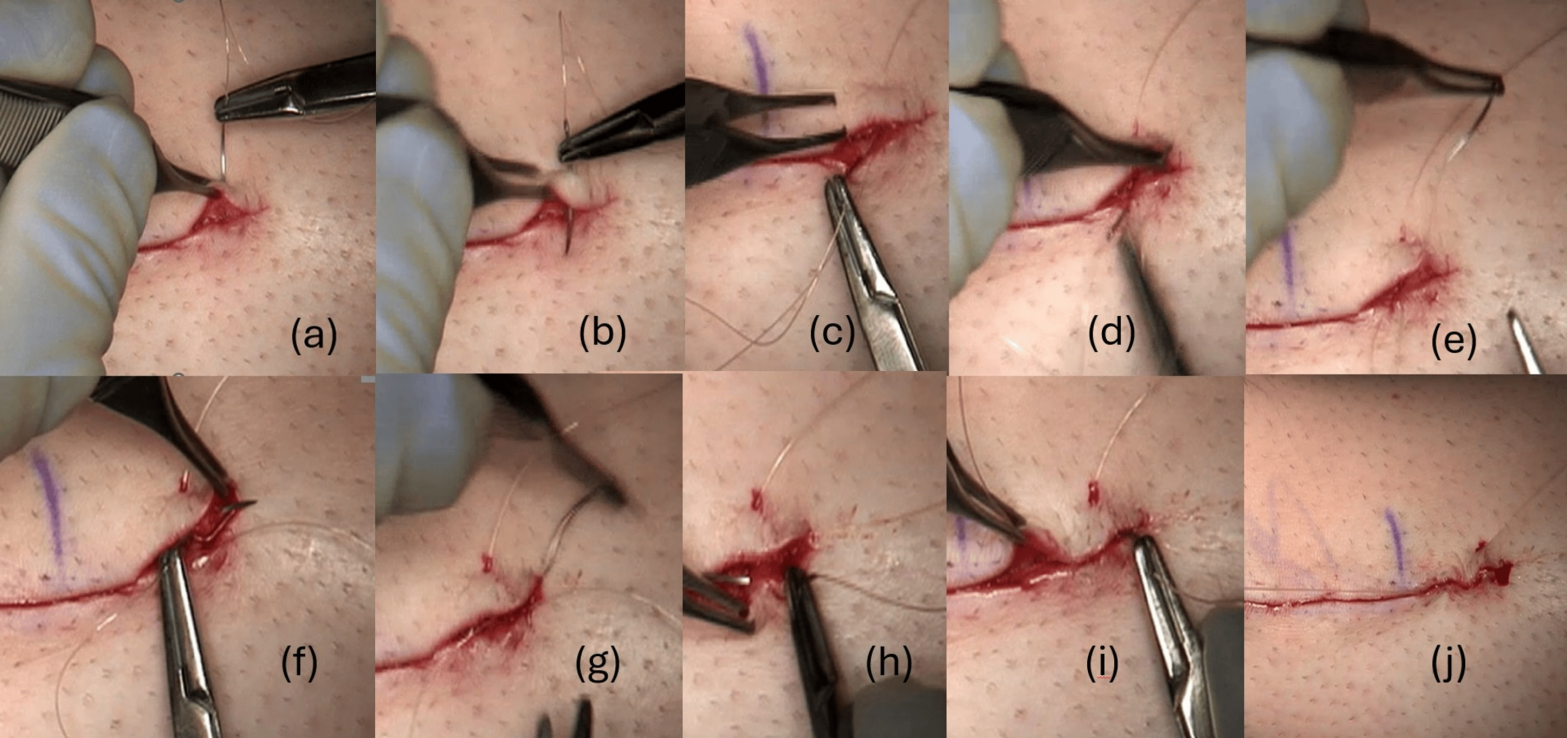
Running subcuticular skin closure using non-barbed absorbable suture with neither knots nor tails The closure starts perpendicular to the incisional wound 1 to 2 cm from the end of the incision (a) and into the wound, deeper in the subcuticular level (b). Second and third throws go out towards the pole of the incision (c-g), with the latter made deep to superficial (f,g). The next three to four throws are done in the standard fashion, going from the pole to the body of the incision (h,i). The suture is then passed back and forth to achieve approximation of the skin edges at the pole (j). This is continued to the other pole, where the reverse of the above is done. As the steri-strips are applied, the suture tails are cut under tension, resulting in no knots and no exposed tail outside of the skin.

**Figure 3:**
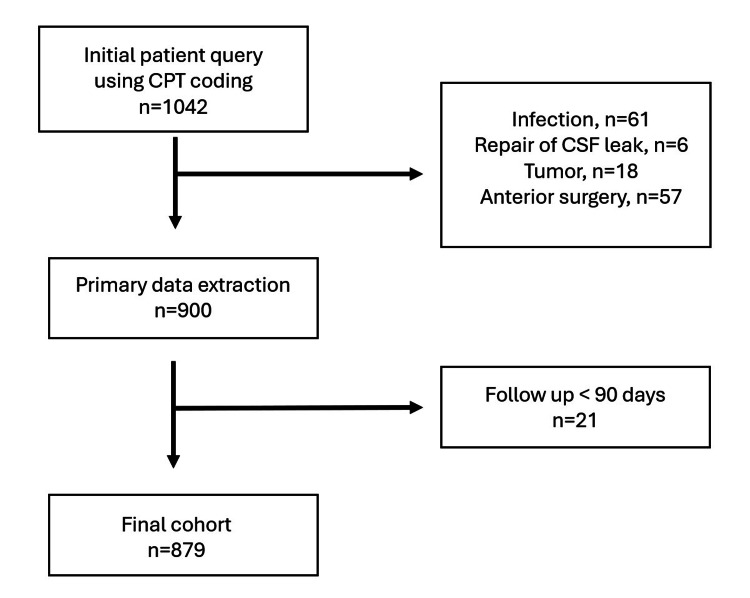
Flowchart of inclusion/exclusion criteria results CPT: Current Procedural Terminology

Inclusion criteria were posterior thoracic and lumbar spine surgery in adult patients (age >21) and skin closure using RSAS, with BK that may be associated with abscesses at poles of incisions (Figure 1) or with KT (Figure 2, Appendices 1, 2). Exclusion criteria were previous infections in the same site, ROR cases for dural repair, oncologic surgery, multiple procedures under the same anesthesia, such as combined anterior-posterior surgeries that would make operative time for the posterior surgery difficult to determine, and less than three months of follow-up (f/u) (Figure 3).

Power analysis

The KT group is comprised of cases closed with the KT method by the senior author (SK) between 2013 to 2023 (n=266). Power analysis using a rate of ROR for deep SSI of 2.5% for the BK group, with significance set at 0.05 and power of 80% suggested that n>600 in the BK group would be needed to demonstrate statistical significance. The BK group is comprised of cases closed using the BK method by three surgeons, A, B, and C, from 2018 to 2020 (n=613).

Medical records review

Demographics, comorbidities, and surgical characteristics were obtained from the electronic medical record. In the KT group, drain data were missing in 15 patients due to the transition to a new electronic medical record; drain data reported for the KT group were from the 251 remaining patients. For the spine division, the common clinical pathway included appropriate preoperative antibiotics, chlorhexidine-based skin prep, and after the skin dried, application of an adhesive incisional skin drape. As a training facility, rotating fellows and residents assist in closing the surgical wound in layers.

Using the medical records review, we identified all patients who had ROR for deep SSI within 12 months after the index surgery. All had ROR within three months of the index surgery and were included in the study. The diagnosis of deep SSI was made by the operating room culture results and/or by the primary surgeon at the time of ROR by finding a disruption of the deep fascial closure with subfascial fluid collection that appeared suspicious for infection.

At ROR for deep SSI, patients underwent irrigation and debridement with retention of instrumentation for those who had them. After a period of four to six weeks of intravenous antibiotics targeted to the organism(s) identified in the positive cultures, patients with implants were placed on chronic oral suppressive antibiotics managed by infectious disease consultants.

Statistical analysis

Descriptive statistics, including mean and standard deviation for continuous variables and frequency and proportion for categorical variables, are reported to characterize the study group. Differences in means between closure groups were compared with independent t tests, whereas differences in proportions were compared with the chi-square or Fisher’s exact test where appropriate. For comparisons with greater than two categories, e.g., levels fused, adjusted standardized residuals were obtained with values greater than two indicating a lack of fit with the null hypothesis of no difference between the groups. Univariate logistic regression was used to quantify the risk of postoperative infection. A multivariate logistic regression model of infection risk was developed using Firth’s bias reduction method. The Firth method addresses potential bias arising from sparse event data. Model coefficients are presented as odds ratios (ORs) with corresponding 95% confidence intervals. All analyses were carried out in R (R Core Team, http://www.r-project.org, R Foundation for Statistical Computing, Vienna, Austria) or MedCalc (www.medcalc.org, MedCalc Software Ltd., Ostend, Belgium).

## Results

The risk for infection was greater in the KT group (n=266) as the KT group had a greater percentage of patients with American Society of Anesthesiologists (ASA) 3 and 4, incidence of smoking history, previous surgery at the same site, number of levels fused, instrumented cases, and longer operating time and hospital stay compared to the BK group (n=613), and the differences were statistically significant. For the other variables, age, sex, BMI, and diabetes history, there were no differences between the two groups. However, the rate of ROR for deep SSI in the KT group (0.38%) was less than that in the BK group (2.6%); the difference was statistically significant (p=0.030) (Table 1).

**Table 1:** Comparisons between cases closed with the KT and BK suture method KT: knotless tailless; BK: buried knots; df: degrees of freedom; V: Cramer’s V; ROR: return to the operating room; SSI: surgical site infection; ASA: American Society of Anesthesiologists. Values are presented as mean (standard deviation) and frequency (proportion) where appropriate. *, **, and *** denote that the adjusted standardized residuals were greater than two in the comparisons of the subgroups, none, 1 level, and 2 or more levels, indicating a lack of fit with the null hypothesis of no difference, i.e., that subgroups none, 1 level, and 2 or more levels were all different between the KT and BK.

Variables	KT (n=266)	BK (n=613)	p-value (df, V)
Age	59.3 (+/- 17.4)	61.2 (+/- 15.2)	0.098
Sex (Male)	131 (49.2%)	322 (52.5%)	0.371 (df=1, V=0.030)
BMI	29.3 (+/- 6.5)	29.0 (+/- 5.8)	0.499
ASA Classes			0.041 (df=3, V=0.097)
ASA 1	15 (5.6%)	41 (6.7%)	0.558 (df=1, V=0.020)
ASA 2	133 (50.0%)	358 (58.4%)	0.021 (df=1, V=0.078)
ASA 3	112 (42.1%)	208 (33.9%)	0.021 (df=1, V=0.078)
ASA 4	6 (2.3%)	6 (1.0%)	0.134 (df=1, V=0.051)
ASA 3 & 4	118 (44.4%)	214 (34.9%)	0.008 (df=1, V=0.090)
Diabetes	59 (22.2%)	123 (20.1%)	0.484 (df=1, V=0.024)
Smoking (current and former)	48 (18.0%)	59 (9.6%)	<0.001 (df=1, V=0.118)
Previous surgery at the same site	88 (33.1%)	97 (15.8%)	<0.001 (df=1, V=0.195)
# Levels fused	3.2 (+/- 3.1)	1.9 (+/- 1.7)	<0.001
None	87 (32.7%)	429 (70.0%)	*
1 level	70 (26.3%)	112 (18.3%)	**
2 or more levels	109 (41.0%)	72 (11.7%)	***
Instrumented fusion cases	165 (62.0%)	182 (29.7%)	<0.001 (df=1, V=0.304)
Case length (minutes)	209.6 (+/- 97.3)	146.2 (+/- 86.2)	<0.001
Drains	157 (62.5%)	234 (38.2%)	<0.001 (df=1, V=0.222)
Hospitalized days	3.3 (+/- 1.9)	2.0 (+/- 2.0)	<0.001
Rate of ROR for deep SSI	1 (0.38%)	16 (2.6%)	0.030 (df=1, V=0.075)

Univariate analysis of infection risk showed that only the odds ratio for diabetes history reached statistical significance (7.4, 95% CI 2.77 - 21.74, p<0.001) (Table 2).

**Table 2:** Univariate analysis of infection risk

Variables	OR (95% CI)	p-value
Age (per 10-year increase)	1.32 (0.95 - 1.91)	0.122
Sex (male)	0.83 (0.31 -2.20)	0.709
BMI (per 10-unit increase)	1.62 (0.77 - 3.15)	0.177
Diabetes	7.40 (2.77 - 21.74)	<0.001
Smoking (current and former)	0.96 (0.15 - 3.47)	0.959
Previous surgery same site	0.23 (0.01 - 1.14)	0.155
# Levels fused (ref: None)		
1 level	2.25 (0.80 - 6.14)	0.122
2 or more	0.31 (0.02 - 1.68)	0.272
Instrumented fusion cases	1.37 (0.51 - 3.62)	0.064
Case length (per 10-minute increase)	1.01 (0.96 - 1.06)	0.643
Drains	2.08 (0.76 – 5.66)	0.154
Hospitalized days (ref: 0-2)		
>3	2.26 (0.85 – 5.99)	0.102
Buried knots	7.10 (1.44 – 128.51)	0.058

Odds ratio for BKs and instrumented fusion reached the threshold of p<0.10. Using multivariate analysis of infection risk, the OR for diabetes history and BKs reached statistical significance (Table 3). Therefore, the BKs method was associated with a higher rate of ROR for deep SSI compared to the KT method.

**Table 3:** Multivariate penalized logistic model of infection risk

Variables	OR (95% CI)	p-value
Buried knots	6.83 (1.59 - 64.29)	0.007
Diabetes	7.33 (2.81 - 20.76)	<0.001
Instrumented fusion cases	1.96 (0.72 - 5.28)	0.186

The deep SSI rates for the three surgeons, A, B, and C, in the BK group were 2.4%, 1.9% and 3.5% respectively, and the comparisons between the surgeons did not reach statistical significance: A vs. B (p=0.75), B vs. C (p=0.28), and A vs. C (p=0.58) (Table 4).

**Table 4:** Individual surgeons' deep surgical site infection rates in the buried knots group rROR: rate of return to the operating room; SSI: surgical site infection; df: degrees of freedom; V: Cramer’s V

Details	rROR for deep SSI	p-value (df, V)
Surgeon A	2.4% (3/124)	n/a
Surgeon B	1.9% (5/261)	n/a
Surgeon C	3.5% (8/228)	n/a
A vs. B	n/a	0.75 (df=1, V=0.016)
A vs. C	n/a	0.58 (df=1, V=0.030)
B vs. C	n/a	0.28 (df=1, V=0.050)

The most common organism in the positive cultures was methicillin-sensitive *Staphylococcus aureus* (MSSA) (nine out of 17), with positive polymicrobial cultures in seven out of 17 cases (Table 5).

**Table 5:** Culture results in patients with deep surgical site infection BK: buried knots; KT: knotless and tailless; *C. diphtheria: Corynebacterium diphtheria; E. coli: Escherichia coli; E. faecalis: Enterobacter faecalis; K. pneumonia: Klebsiella pneumoniae;* MRSA: methicillin-resistant *Staphylococcus aureus*; MSSA: methicillin-sensitive *Staphylococcus aureus*; *P. acnes: Propionibacterium acnes; P. aeruginosa: Pseudomonas aeruginosa; Staph. epi: Staphylococcus epidermidis*

Details	Organism
BK1	MSSA
BK2	C. diphtheria, Staph. epi
BK3	MSSA, *K. pneumoniae*
BK4	P. acnes
BK5	P. aeruginosa
BK6	MSSA
BK7	*E. coli, E. faecalis, *MRSA*, K. pneumoniae, Staph. epi*
BK8	P. aeruginosa
BK9	MSSA, P. aeruginosa, C. diphtheria
BK10	MSSA, Morganelli morganii
BK11	E. Coli, C. diphtheria, E. faecalis, Streptococcus
BK12	MSSA
BK13	MSSA
BK14	Serratia marcescens
BK15	MSSA, *Peptostreptococcus*
BK16	MSSA
KT 1	P. acnes

## Discussion

We reject the null hypothesis that the rate of ROR for deep SSI is the same for the KT and BK methods. We believe that our findings are significant because the KT group had a higher risk for SSI in seven of the 11 variables assessed (ASA classes, smoking history, previous surgery at the same site, number of levels fused, instrumented fusion cases, longer case length, and hospital stay) and no difference in the other three variables (age, BMI, and diabetes history). Yet, the rate of ROR for deep SSI in the KT group was less than in the BK group.

A literature search for skin closure in spine surgery and SSI (Table 6) found studies that showed an increased rate of SSI with staples compared to adhesive [8], adhesive with polymer [9], continuous nylon sutures [10], and RSAS [11] and three that did not find any differences between staples and other methods [12-14]. No difference in SSI rates was seen between suture vs. adhesive strips [15] and different suture methods [16], but one study found decreased SSI with subcuticular barbed suture versus absorbable sutures [17], while a second did not [18]. Subcuticular closure using a barbed suture can also be knotless and tailless, though barbed sutures are used more often in the fascial and subcutaneous levels [19]. In a direct comparison between subcuticular closure using non-barbed absorbable monofilament or barbed absorbable suture or transdermal nylon sutures for total knee arthroplasty, subcuticular wound closure with absorbable monofilament had fewer complications than the barbed suture, more aesthetics than the transdermal suture, and was more cost-effective than both [20]. The current evidence for using barbed sutures at the subcuticular level is inconclusive.

**Table 6:** Literature search for skin closure in spine surgery and surgical site infection pros: prospective; retro: retrospective; RCT: randomized controlled trial; Inc: increased; ND: no difference; RSAS: running subcuticular skin closure using non-barbed absorbable suture

Authors	Study Type	Type of Surgery	Risk for SSI
Ando et al. (2014) [8]	Pros	Spine	Inc w/staples vs. skin adhesive
Johnston et al. (2020) [9]	Retro	Spinal fusion	Inc w/staples vs. adhesive + polymer
Shani et al. (2020) [10]	Retro	Posterior spine	Inc w/staples vs. continuous nylon
Kim et al. (2025) [11]	Retro	Posterior spine	Inc w/staples vs. RSAS
Akshay et al.(2024) [12]	Retro	Obese, posterior lumbar one-level fusion	ND staples, absorbable suture
Molliqaj et al. (2024) [13]	Retro	Posterior spine	ND staples, sutures, adhesives, or polymer
Romagna et al. (2025) [14]	RCT	Non-instrumented posterior lumbar	ND staples, intracutaneous sutures
Nunez-Moreno et al. (2025) [15]	Retro	Posterior spinal fusion	ND suture, adhesive strip
Mostofi et al. (2022) [16]	Retro	Lumbar spine	ND different suturing methods
Shi et al. (2022) [17]	RCT	Long segment posterior lumbar spine	Inc w/subcuticular w/ 3-0 Vicryl vs. barbed suture
Chen et al. (2018) [18]	Retro	Thoracolumbar fracture	ND interrupted 4-0 Vicryl vs. barbed suture

Limitations

First, our study compares results from one surgeon with three other surgeons and may be subject to unaccounted confounders. However, because of common clinical pathways to prevent SSI and because the surgical wounds were closed primarily by rotating fellows and residents, the wound closure other than the subcuticular layer should be similar in the two groups. Second, the study groups are noncontemporary. To have the power to see the difference between the groups, the KT group was collected over a longer time period. Because of this, it is possible that improved and standardized SSI prevention strategies implemented at this institution may have affected the rate of ROR for deep SSI. We do not believe that this is the case, as there have not been any significant changes to the clinical pathways, and the 2.6% deep SSI rate seen in the BK group is in line with the infection rates seen in the literature. Third, this is a retrospective study, and we could not assess other possible confounders, such as lower albumin and the severity of risk factors, such as diabetes. Fourth, this is a single division, single institution experience.

## Conclusions

For running subcuticular skin closure using absorbable suture, our study suggests that elimination of BKs by using the overlapping knotless and tailless method may decrease deep SSI rates in posterior thoracic and lumbar spine surgery. Further studies are warranted to study the effect of the KT method on deep SSI rates and to identify the optimal wound closure method.

## Appendices

Appendix 1 

Appendix 2
